# The quantitative measurements of choroidal thickness and volume in diabetic retinopathy using optical coherence tomography and optical coherence tomography angiography; correlation with vision and foveal avascular zone

**DOI:** 10.1186/s12886-021-02178-w

**Published:** 2022-01-03

**Authors:** Fariba Ghassemi, Sahar Berijani, Ameneh Babeli, Houshang Faghihi, Alireza Gholizadeh, Siamak Sabour

**Affiliations:** 1 Eye research center, Farabi Eye Hospital, Tehran University of Medical Sciences, Qazvin Square, Tehran, IR 1336616351 Iran; 2 Retina & Vitreous Service, Farabi Eye Hospital, Tehran University of Medical Sciences, Tehran, IR Iran; 3 Department of Clinical Epidemiology, School of Public health and Safety, Safety Promotion and Injury Prevention Research Centre, Shahid Beheshti University of Medical Sciences, Tehran, IR Iran

**Keywords:** Choroidal thickness, Choroidal thickness measurement, Choroidal volume, Diabetes, Diabetic retinopathy, Diabetic macular edema, Foveal avascular zone, Semi-automated choroidal thickness measurement

## Abstract

**Background:**

To represent choroidal thickness (CT) and choroidal volume (CV) databases in diabetic retinopathy (DR) patients and healthy control participants using optical coherence tomography (OCT) and enhanced depth imaging OCT (EDI-OCT). No study had evaluated CT at all main stages of diabetic retinopathy in a single study.

**Methods:**

The study included 176 eyes from 93 patients (39–80 years old; 42% females) who were divided into three groups based on DR severity and normal control group: 39 eyes no DR, 64 eyes NPDR, 33 eyes PDR, and 40 eyes normal control. The CT and CV were measured and statistically analyzed. Intra-observer and inter-observer coefficients of repeatability were calculated.

**Results:**

Subfoveal CT showed persistent thinning from normal group (322.50 ± 69.24) to no-diabetic retinopathy (NDR, 308.33 ± 74.45) to nonproliferative diabetic retinopathy (NPDR, 283.45 ± 56.50) group and then thickening as the patient progressed to proliferative diabetic retinopathy (PDR, 295.17 ± 95.69) (*P* = 0.087). A significant difference was found between the control group and the NDR, NPDR, and PDR groups in nearly all CT and CV of Early Treatment Diabetic Retinopathy Study macular subfields. Fasting blood sugar (FBS = 189.08 ± 51.3 mg/dl) and diabetes mellitus (DM) duration (13.6 ± 6.5 years) had no noticeable effect on CT. In patients with diabetes, the best-corrected visual acuity (BCVA), diabetic macular edema (DME), and foveal avascular zone (FAZ) were not affected by CT and CV.

**Conclusions:**

The choroidal thickness decreases from the early stages of diabetic retinopathy up to the NPDR stage, with a subsequent modest rise in CT during the PDR stage. There was no correlation between FBS, diabetes duration, BCVA, DME, and FAZ, and CT.

## Background

Diabetes mellitus (DM) has emerged as one of the world’s most critical challenges. Diabetes is a metabolic disease affecting the vascular system. While the main changes in diabetic eyes occur in the retinal vasculature, some changes have also been detected in the choroidal layer, which supplies the outer retina [1–3]. Considering that the choroid is the most vascularized and metabolically active tissue in the eye, its pathologic and clinical involvement may be expected in diabetes [3].

Several studies have indicated that the choroid is a key participant in the pathogenesis of diabetic retinopathy (DR) and diabetic macular edema (DME) due to changes in the blood retinal barrier (BRB) [1, 2].

The concept of diabetic choroidopathy was first introduced at 1985 [1]. Histologic studies showed that the choroid of persons with DME often displays increased vessel tortuosity, with microaneurysm and narrowing, hyper-cellularity, vascular loops, microaneurysms; choriocapillaris (CC) drop-out, neovascularization, and sinus-like configurations between choroidal lobules [1, 4–6].

In addition, studies using indocyanine green angiography (ICG) showed filling delay or defects in the choriocapillaris, sacular dilatations, microaneurysms in the choriocapillaris, and choroidal neovascularization in up to 50% of the nonproliferative diabetic retinopathy (NPDR) cases [7–9].

High resolution scans of the retina and choroid using newer spectral domain optical coherence tomography (SD-OCT) techniques such as enhanced depth imaging (EDI) provide additional structural and quantitative information in the characterization of diabetic retinopathy, particularly DME, and because it is noninvasive, it has surpassed the use of invasive fluorescein angiography (FA) for follow-up [10–12].

Choroidal thickness (CT) has been used as a surrogate marker for choroidal health evaluation. CT studies in diabetes have produced conflicting findings; the studies suggested choroidal thickening [2, 13, 14], thinning [10, 15], and no change [10, 16] in eyes with diabetic retinopathy. According to some of these studies, the choroid in DME is thinner than normal [2, 10, 17]. An observation that might be attributed to the alterations in the underlying choroid, which might have a role in the onset or advancement of the disease process [2, 10, 17].

To the best of our knowledge, no study had evaluated CT at all main stages of diabetic retinopathy in a single study at the time of writing this article. The current research aimed to assess retinal thickness (RT), CT, and choroidal volume (CV) and their correlation with foveal avascular zone (FAZ) and best corrected visual acuity (BCVA) in diabetic patients with no diabetic retinopathy (NDR), nonproliferative diabetic retinopathy (NPDR), and proliferative diabetic retinopathy (PDR), with or without DME, and normal volunteers in the Iranian community.

## Methods and material

This cross-sectional study was conducted in Farabi Eye Hospital, a tertiary university eye centre in Tehran, IR Iran, from January 2015 through December 2019. Institutional ethics committee of Tehran University of Medical Sciences approved the protocol and a written informed consent was obtained from the participants. The study adhered to the tenets of the Declaration of Helsinki. A total of 105 patients were recruited. Twelve of the eligible patients refused to provide informed consent, so their data was excluded. The examinations and imagings were done at the first day visit.

### Patient eligibility

For the study, naïve diabetic patients with a history of diabetes mellitus for more than 10 years were recruited, while healthy volunteers participated as controls. Inclusion criteria were best-corrected visual acuity of 20/20 for normal cases and refractive error between − 3 and + 1 D spherical equivalent in all groups. Exclusion criteria were significant media opacity precluding high-quality imaging, and existing of motion and blinking artifact on the images. The advanced PDR cases with poor quality images were excluded. Normal volunteers did not have any ocular or systemic disorder. Previous treatment with focal or panretinal laser photocoagulation, intravitreal anti-vascular endothelial growth factor (VEGF), steroid and/or potentially toxic drugs to the retina, choroid and/or optic nerve, previous ocular surgeries other than uncomplicated phacoemulsification and intraocular lens implantation, inflammatory diseases or active or recent infection (ocular and/or systemic), systemic treatment with corticosteroids, immunosuppressive drugs or biologic therapies, pregnancy, and uncontrolled hypertension were in the exclusion criteria list. Hypertension control was defined as systolic blood pressure below or equal to 140 mmHg and diastolic blood pressure below or equal to 85 mmHg in the clinic and in the sitting position.

Full ophthalmologic examination including clinical history, past medical history, best-corrected visual acuity (BCVA) based on Logarithm of the Minimum Angle of Resolution (LOGMAR), biomicroscopy of the anterior segment using a slit lamp, Goldman applanation tonometry, and indirect ophthalmoscopy were performed. The fasting blood sugar (FBS) level was measured in all diabetic participants, but the glycosylated hemoglobin (HbA1c) level was not assessed in some of the participants. Diabetes was diagnosed among all study participants using American Diabetes Association guidelines, and they were all under diabetes treatment. The patients with diabetes were divided into three groups depending on the degree of DR according to the Early Treatment Diabetic Retinopathy Study (ETDRS) criteria as NDR, NPDR, and PDR. Clinical examination and SD-OCT, enhanced depth imaging (EDI-OCT) (Heidelberg Corporation, Germany) and optical coherence tomography angiography (OCTA) (AngioVue OCT-A system version 2018,0,0,18 (RTVue XR Avanti; Optovue, USA- pre-release version: 2016.1.0.23- beta) imagings were performed at the same day between 8:00 am and 2:00 pm. All OCT scans were conducted under standardized mesopic lighting conditions by the same experienced operator. By EDI-OCT, a 24 horizontal line raster scan (30^o^ × 25^o^, 9.2 mm × 7.6 mm) centered on the fovea was performed, with 24 frames averaged in each OCT B-scan to improve the image quality. Two professional image readers (FG, SB) checked and assessed all of the OCT images to ensure that the scans were of sufficient clarity to adequately visualize the choroid–scleral boundary on every B-scan. If the scans were of inadequate quality, they were immediately repeated. The CT and CV measures were obtained using the EDI-OCT, and the FAZ by the OCTA.

### CT and CV measurements using SD-OCT

**Fig. 1:**
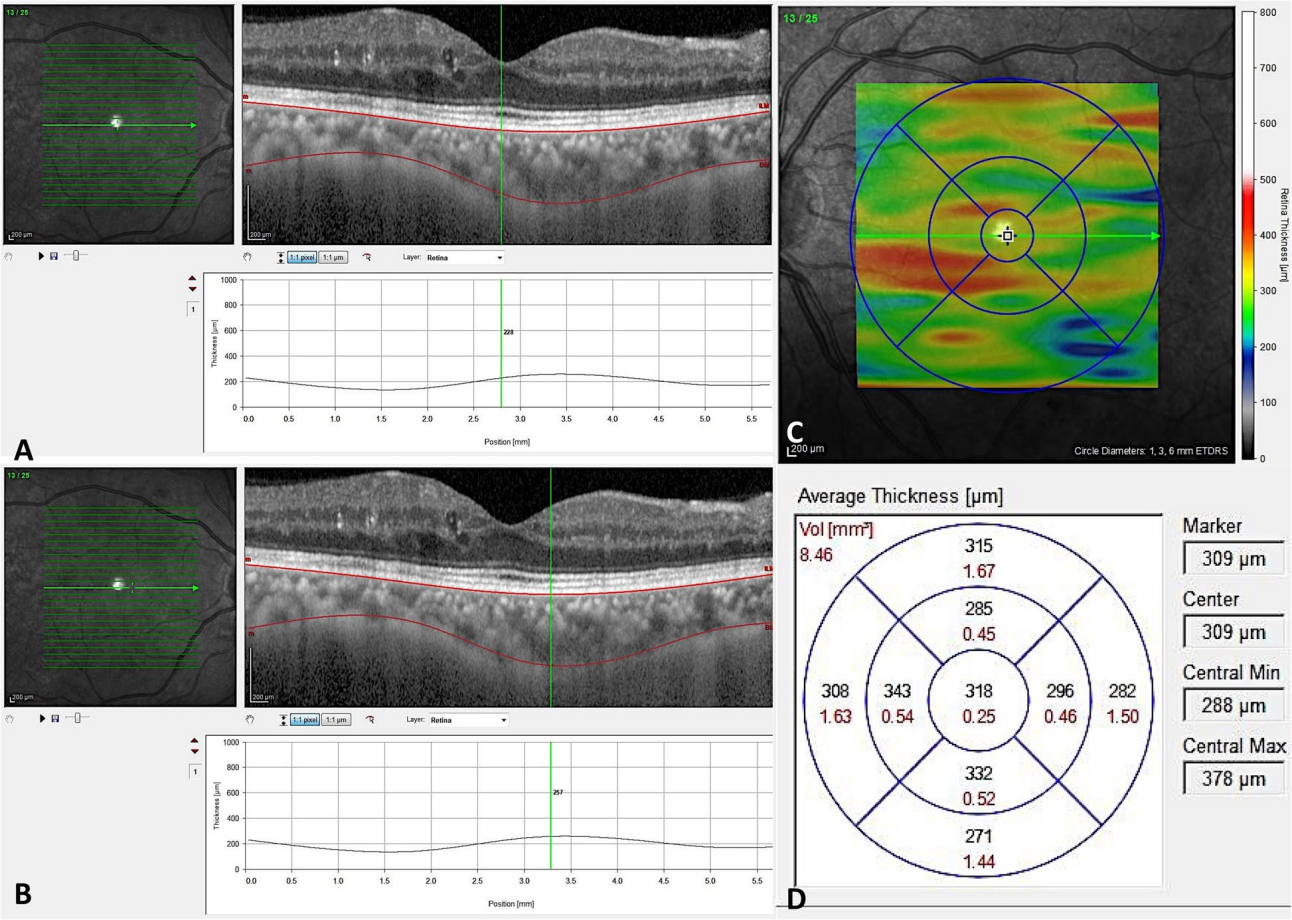
Choroidal thickness measurement in horizontal line crossing foveola (**A** and **B**) and choroidal thickness mapping by sequential thickness measurement in 24 grid lines (**C** and **D**)

The choroid was semi-automatically segmented using two manually located slabs from the outer edge of the hyper-reflective retinal pigment epithelial-Bruch’s membrane complex (RPE-Bruch’s) to the inner edge of the sclera (sclerochoroidal (SC) junction) (Fig. 1) as already defined [18]. For the creation of choroidal thickness maps, the measurements were performed on at least 24 horizontal raster lines taken from the fovea-centered macular region. Automated image segmentation methods have been described, and evaluated on clinical populations [12–17].

Two observers (SB, AB) managed the segmentations. We selected the retinal thickness map analysis protocol to display numerical averages of the thickness and volume measurements for each of the 9 subfields, as defined by the Early Treatment Diabetic Retinopathy Study (ETDRS). It was described as 3 concentric circles defining 9 macular subsegments (1 mm center, 3 parafoveal area, and 6 mm perifoveal area at nasal, temporal, superior and inferior parts). In addition, the ETDRS thickness map was converted into 3 circular regions of 1, 3, and 6 mm where the outer sections contained the previous sections [13–17]. The CV and CT maps were determined and the measures recorded (Fig. 1). Inferior quality scans (< 70/100) have been discarded. In addition, pointy choroidal thicknesses measurements in five parts of fovea (subfoveal CT-SFCT), and o.5 and 1.5 mm nasal (N0.5, N1.5 mm) and temporal (T0.5, T1.5 mm) to the fovea were achieved on the scan crossing the center of the fovea by semi-manual technique.

In order to confirm the validity of the measurements, the main trained graders (SB and AB) measured the results twice for the first 40 eyes. Both intra-grader and inter-grader reliability was high with intra-class correlation coefficient (ICC) of 0.959 and 0.935, or greater respectively.

### Foveal avascular zone (FAZ) measurement using Optovue

AngioVue OCTA imaging using the split-spectrum amplitude decorrelation angiography (SSADA) algorithm, was performed [18]. This instrument operates at 840-nm wavelength and performs 70,000 A-scans per second to acquire OCTA of two repeated horizontal and vertical B-scans of 3 × 3 mm (304 × 304 pixels) in the transverse dimension. Low quality scans have been omitted and replicated until scans of good quality have been achieved.

The automated segmentation with the preset settings was used for all measurements. In brief, a non-flow area measurement of the superficial and deep vascular plexus of the enface projection was performed. The operator manually fine-tuned the plane to optimize the visualization of the retinal capillary bed. Upon clicking on the centre of the FAZ, the software automatically calculated the area [19, 20].

### Statistical analysis

All quantitative variables were reported as mean with standard deviation after confirming normality of distribution with the Kolmogorov- Smirnov test and histogram. The median with the range was documented for variables with skewed deviation. Parametric and nonparametric analysis were performed accordingly.

All statistical analyses were performed using statistical software (SPSS Version 21; SPSS, Inc., Chicago, IL, USA). Student’s t-test or Analysis of Variance (ANOVA) (dunnett’s test for post-hoc analysis) was used to compare choroidal thicknesses between groups. In this study collinearity for different variables was checked. The correlation of DME to CT was evaluated after adjusting for age, gender, hypertension, hyperlipidemia, and FBS by binary logistic regression analysis (including all correlated factors in partial correlation analysis in the model). *P* values less than 0.05 were considered statistically significant.

## Results

### General characteristics

A total of 176 eyes of 93 patients were included in the study (42.6% women, 52.3% right eyes, mean age 57.3 ± 8.2 years, range 39–80 years). Mean FBS was 189.08 ± 51.3 mg/dl in the patients with diabetes. Mean DM duration in patients with diabetes was 13.6 ± 6.5 years (1–30 years). The healthy group included 40 eyes and the group with diabetes comprised 136 eyes. Based on the DR severity scale, the group with diabetes were classified as 39 eyes with NDR, 64 eyes with NPDR (40 eyes with mild to moderate NPDR and 24 eyes with severe NPDR), and 33 eyes with PDR (20 early PDR and 13 eyes with high-risk characteristic PDR including preretinal and vitreous hemorrhage) and compared with 40 normal participants’ eye. DME was present in 62.7% of NPDR and 57.9% of PDR patients. The general characteristics of each group are summarized in Table 1.

**Table 1:** Demographic data of the studied normal participants and diabetic patients

Groups	Control (40 eyes) (M-range) (μm)	NDR (39eyes) ((M-range) (μm)	NPDR (64eyes) ((M-range) (μm)	PDR (33eyes) ((M-range) (μm)
**Age (Y)**	50 ± 0.7	59.2 ± 1.1	58.3 ± 1.1	59.1 ± 1.7
**OD(%)**	(50) 20	(51.3) 20	(51.6) 33	(57.6) 19
**Sex Male (%)**	(60) 24	(27.2) 11	(42.2) 27	(39.4) 13
**BCVA (LOGMAR)**	0.02 ± 0.01	0.09 ± 0.01	0.41 ± 0.11	0.59 ± 0.07
**The last FBS** (mg/dl)	–	158.4 ± 5.7	189.7 ± 8.3	202.1 ± 7.3
**Duration of diabetes mellitus (Y)**	–	12.3 ± 1.1	12.9 ± 0.7	16.5 ± 1.1
**Hypertension number (%)**	2 (5%)	16 (41%)	27 (42%)	17 (51.5%)
**Hyperlipidemia number (%)**	0 (5%)	15 (38.5%)	32 (50%)	16 48.5%)
**DME**	0 (0%)	0 (0%)	40 (62.5%)	19 (57.6%)

**BCVA Best corrected visual acuity, *DME* Diabetic macular edema, *FBS* Fasting blood sugar, *NDR* No diabetic retinopathy, *NPDR* Nonproliferative diabetic retinopathy, *PDR* Proliferative diabetic retinopathy

As expected, the groups had different BCVA. The groups did not differ significantly in spherical equivalent and intraocular pressure. Hyperlipidemia and hypertension were more common in the groups with diabetes compared to the healthy group (*p* < 0.001). The hypertension was under systemic control in all groups.

### Choroidal thickness (CT) measurements by OCT

**Fig. 2:**
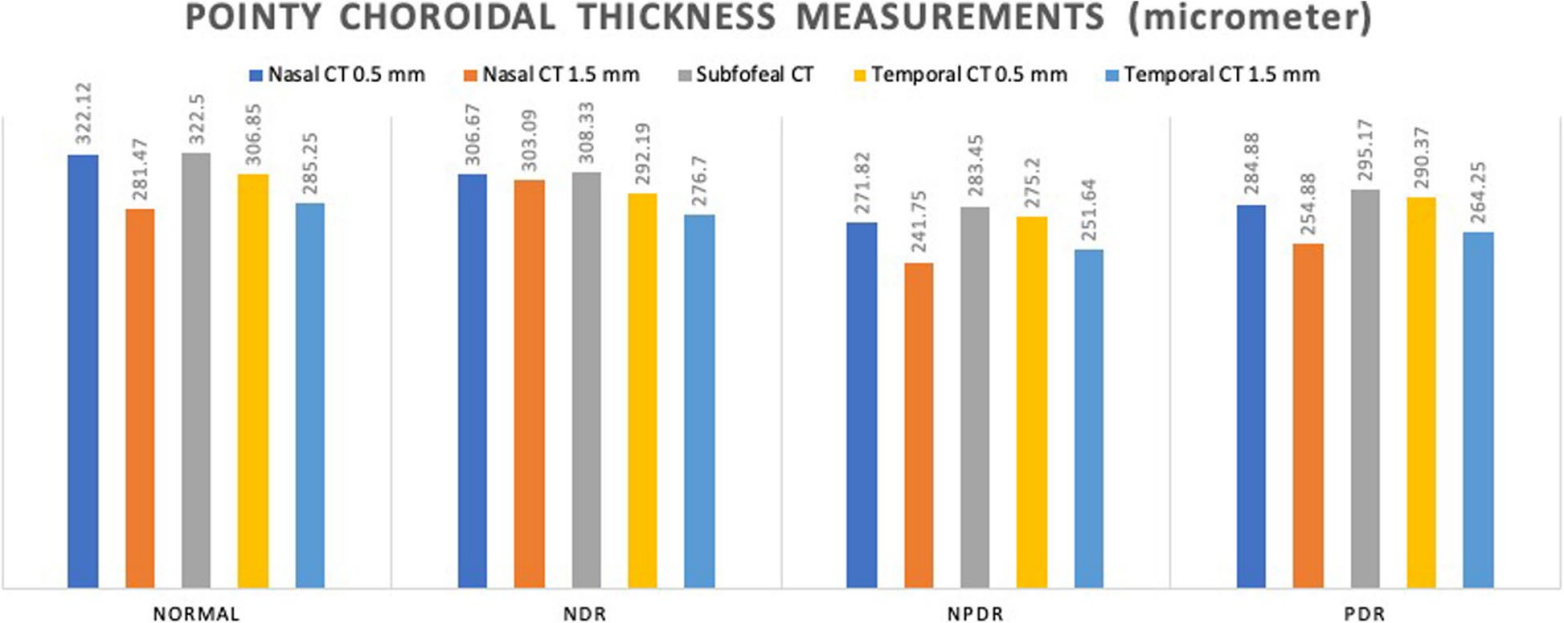
Pointy choroidal thickness measurements using optical coherence tomography scanned across the fovea center

Generally, patients with diabetes had less choroid thickness (CT) than healthy participants at subfoveal (SFCT) and N0.5 mm CT (295.1 ± 73.1 μm vs. 322.5 ± 69.2 μm; *p* < 0.037 and 293.3 ± 80.1 μm vs. 322.1 ± 68.9 μm; *p* < 0.041, respectively) (Table 2). Figure 2 show pointy CT at 0.5 mm and 1.5 mm from the fovea on the nasal and temporal sides on the line crossing the center of the fovea to the inferior disk margin in various diabetes types.

**Table 2:** Comparison between choroidal thickness of normal participants and patients with diabetes

Groups	Control (40) (M ± SD) (μm)	DR (136) (M ± SD) (μm)	***P***-value
CT			
**FT (median)**	227.7 ± 26.8	262.8 ± 88.5	0.015
**CMT**	272.5 ± 23.0	297.9 ± 74.9	0.036
**SFCT**	322.5 ± 69.2	295.1 ± 73.1	0.037
**CT N0.5 mm**	322.1 ± 68.9	293.3 ± 80.1	0.041
**CT N1.5 mm**	281.4 ± 61.1	265.8 ± 81.1	0.260
**CT T0.5 mm**	306.8 ± 70.8	289.7 ± 70.2	0.178
**CT T1.5 mm**	285.3 ± 58.7	268.6 ± 76.6	0.208

**Choroidal thickness, *FT* Foveal thickness, *DR* Diabetic retinopathy, Subfoveal choroidal thickness

**Fig. 3:**
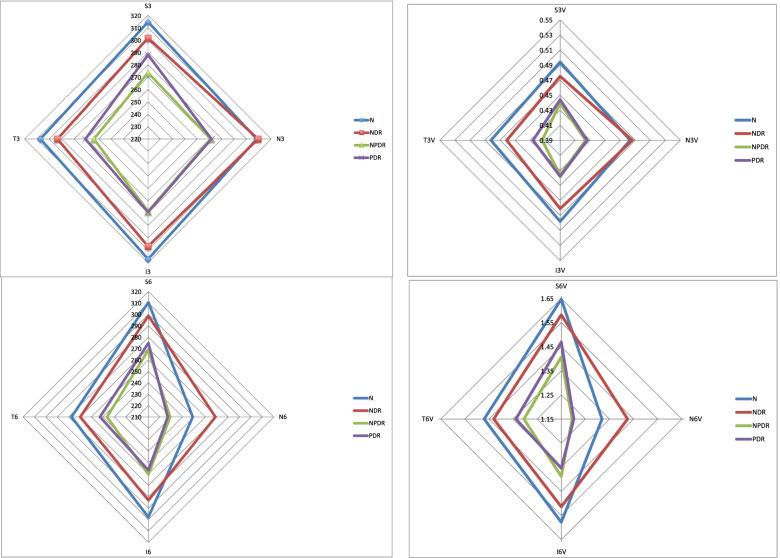
The radar diagram (**A**-**D**) of choroidal thickness (**A**, **B**) and choroidal volume (**C**, **D**) map at 3 mm and 6 mm EDTRS grid subfields (showing variation of the amounts taken by SD OCT)

Measurements revealed the thickest point of choroid was at SFCT (308.33 ± 74.45 μm), followed by nasal (N0.5 = 306.67 ± 80.10 μm, N1.5 = 303.09 ± 80.43 μm) and then temporal (T0.5 = 292.19 ± 72.13 μm, T1.5 = 276.70 ± 77.68 μm) dimensions in NDR. In NPDR, the choroid was thickest in subfoveal location (283.45 ± 56.50 μm) and the thinner at N1.5 (241.75 ± 55.85 μm) and T1.5 mm (251.64 ± 65.33 μm) points (Fig. 2). The thinnest part was in the N1.5 (241.75 ± 55.85 μm) location. Although the choroidal thickness map in PDR showed less than normal averages, the thicknesses have been comparable to NPDR phase (Table 3 and Fig. 3). The choroidal thicknesses have a declining trend from normal to NDR and then to NPDR. In the PDR phase, choroid thickening was shown in all subfields relative to the NPDR phase. In 3 of 5 choroidal pointy measurements at SFCT (283.45 ± 56.50 μm vs. 322.50 ± 69.24 μm, *P* = 0.037), N0.5(271.82 ± 52.51 μm vs. 322.12 ± 68.99 μm, *p* = 0.023), N1.5(241.75 ± 55.85 μm vs. 281.47 ± 61.02 μm, *P* = 0.031), significant differences (*P* < 0.05) were detected between the NPDR and healthy groups, respectively.

**Table 3:** Subfields choroidal thickness of the normal participants and patients with diabetes

Groups	Control (40 eyes) (M-range) (μm)	NDR (39eyes) ((M-range) (μm)	NPDR (64eyes) ((M-range) (μm)	PDR (33eyes) ((M-range) (μm)	***P***-value
CT					
**CCT 1 mm**	322.5 ± 61.1	306.2 ± 63.1	280.3 ± 48.1	285.9 ± 82.3	0.007
**S3**	314.6 ± 57.6	301.7 ± 56.8	273.6 ± 44.4	288.0 ± 81.3	0.010
**I3**	308.2 ± 57.6	309.3 ± 62.1	270.8 ± 50.6	271.5 ± 87.5	0.002
**N3**	317.1 ± 60.9	306.9 ± 62.5	278.4 ± 63.9	279.2 ± 81.4	0.004
**T3**	307.1 ± 56.9	293.3 ± 62.1	263.9 ± 47.7	270.8 ± 65.9	0.001
**S6**	310.5 ± 66.1	298.6 ± 51.7	268.7 ± 47.1	274.8 ± 78.4	0.002
**I6**	248.9 ± 54.5	268.8 ± 58.3	229.1 ± 51.6	226.9 ± 80.1	0.002
**N6**	297.9 ± 61.7	283.1 ± 63.3	260.5 ± 62.1	256.7 ± 68.8	0.003
**T6**	277.5 ± 49.4	269.8 ± 56.5	246.3 ± 47.6	252.5 ± 56.8	0.003
**ATSMCT**	319.4 ± 10.6	298.3 ± 12.9	284.9 ± 6.7	291.6 ± 17.7	0.113

**ATSMCT Average total submacular choroidal thickness, *CCT* Central choroidal thickness at 1 mm around fovea, *CT* Choroidal thickness, I3 inferior- 3 mm, *I6* Inferior-6 mm, *N3* Nasal- 3 mm, Nasal- 6 mm, *S3* Superior- 3 mm, *S6* Superior-6 mm, N6: *T3* Temporal-3 mm, *T6* Temporal-6 mm

***NDR No diabetic retinopathy, *NPDR* Nonproliferative diabetic retinopathy, *PDR* Proliferative diabetic retinopathy

Table 3 displays the mean CT map data in the groups in 9 EDTRS subfields. Significant differences between the healthy controls and the NDR, NPDR, or PDR groups were found in all subfields (*P* < 0.05- Table 3), respectively; with the exception of the average total submacular CT (ATSMCT as control = 319.4 ± 10.6 μm, NDR = 298.3 ± 12.9 μm, NPDR = 284.9 ± 6.7 μm, and PDR = 291.6 ± 17.7 μm, *P* = 0.113**)**.

Table 4 shows the mean CV in 9 EDTRS subfields in the study groups. Significant differences (Table 4) between groups were found in all sub-fields except S3 and I3. The volumes showed the same harmony as CT that decreased from normal to NDR and then to NPDR and but instead increased in the PDR group. CV(s) in PDR never reached the amounts in normal group in comparison (Fig. 3).

**Table 4:** Subfields choroidal volumes of the normal participants and patients with diabetes

Groups	Control (40 eyes) (M-SD) (μm)	NDR (39eyes) ((M-SD) (μm)	NPDR (64eyes) ((M-SD) (μm)	PDR (33eyes) ((M-SD) (μm)	***P***-value
CV					
**CCV (1 mm)**	0.253 ± 0.047	0.239 ± 0.048	0.220 ± 0.037	0.224 ± 0.064	0.006
**S3CV**	0.494 ± 0.089	0.475 ± 0.088	0.439 ± 0.101	0.444 ± 0.131	0.065
**I3CV**	0.483 ± 0.089	0.486 ± 0.097	0.424 ± 0.079	0.426 ± 0.138	0.294
**N3CV**	0.498 ± 0.095	0.481 ± 0.098	0.436 ± 0.100	0.438 ± 0.127	0.004
**T3CV**	0.482 ± 0.090	0.461 ± 0.097	0.414 ± 0.075	0.426 ± 0.104	0.001
**S6CV**	1.647 ± 0.349	1.582 ± 0.274	1.407 ± 0.280	1.469 ± 0.420	0.002
**I6CV**	1.319 ± 0.288	1.425 ± 0.309	1.195 ± 0.272	1.202 ± 0.424	0.002
**N6CV**	1.578 ± 0.327	1.514 ± 0.322	1.388 ± 0.323	1.354 ± 0.358	0.002
**T6CV**	1.470 ± 0.262	1.430 ± 0.299	1.305 ± 0.252	1.338 ± 0.300	0.006
**ATSMCV**	8.13 ± 0.27	8.21 ± 0.21	7.13 ± 0.15	7.25 ± 0.39	0.001

** *ATSMCV* Total submacular choroidal volume, *CCV* Central choroidal volume (1 mm), *I3CV* Inferior-3 mm, *I6CV* Inferior-6 mm, *N3V* Nasal- 3 mm, *N6CV* Nasal- 6 mm, *S3CV* superior- 3 mm, *S6CV* Superior-6 mm, *T6CV* Temporal-6 mm, *T3CV* Temporal-3 mm

*** *NDR* No diabetic retinopathy, *NPDR* Nonproliferative diabetic retinopathy, *PDR* Proliferative diabetic retinopathy

We noticed no differences in the SFCT (322.50 ± 69.24 μm vs. 308.33 ± 74.45 μm, *P* = 0.300), central macular thickness (CMT, 272.5 ± 22.95 μm vs. 256.94 ± 22.05 μm, *P* = 0.550) and foveal thickness (FT, 247.00 vs. 243.00 median per μm, *P* = 0.876) between normal and NDR groups (Post-HOC analysis-Dunnett’s test). Nonetheless, in comparison with normal cases, the main decrease in these parameters occurred in the NPDR group (Table 5).

**Table 5:** Foveal Thickness, foveal avascular zone and Choroidal thickness in different study groups (ANOVA) normal participants and patients with diabetes. Post Hoc analysis of subfields choroidal thickness of the studied participants (*Dunnett test (2-sided))

Groups	Control (40 eyes) (M-SD) (μm)	NDR (39eyes) ((M-SD) (μm)	NPDR (64eyes) ((M-SD) (μm)	PDR (33eyes) ((M-SD) (μm)	***P***-value (ANOVA)
FT (median, range)	247.00 (226–292)	243.00 (183–290) 0.876*	281.00 (201–664) < 0.001*	284.00 (170–499) 0.041*	< 0.001
CMT (Mean)	272.5 ± 22.95	256.94 ± 22.05 *0.550	320.03 ± 89.43 0.003*	305.43 ± 65.50 0.079*	< 0.001
FAZs	0.28 ± 0.09	0.33 ± 0.11 *0.405	0.35 ± 0.18 0.053*	0.37 ± 0.10 0.003*	0.009
FAZd	0.31 ± 0.08	0.38 ± 0.18 *0.550	0.50 ± 0.26 < 0.001*	0.52 ± 0.20 < 0.001*	< 0.001
**SFCT**	322.50 ± 69.24	308.33 ± 74.45 0.300*	283.45 ± 56.50 0.037*	295.17 ± 95.69 0.299*	0.087
**N0.5 CT**	322.12 ± 68.99	306.67 ± 80.10 0.985*	271.82 ± 52.51 0.023*	284.88 ± 98.37 0.178*	0.022
**N1.5 CT**	281.47 ± 61.02	303.09 ± 80.43 0.178*	241.75 ± 55.85 0.031*	254.85 ± 92.76 0.279*	< 0.001
**T0.5 CT**	306.85 ± 70.82	292.19 ± 72.13 *0.985	275.20 ± 54.76 0.122*	290.37 ± 84.87 0.840*	0.182
**T1.5 CT**	285.25 ± 58.77	276.70 ± 77.68 0.951*	251.64 ± 65.33 0.097*	264.25 ± 78.23 *0.589	0.047

*CT* Choroidal thickness, *CMT* Central macular thickness, *SFCT* Subfields choroidal thickness, *CT N* Choroidal thickness at nasal side of fovea (0.5 and 1.5 mm from fovea), *CT T* Choroidal thickness at temporal side of fovea (0.5 and 1.5 mm from fovea), *FAZ (s and d)* Foveal avascular zone (superficial and deep)

*NDR* No diabetic retinopathy, *NPDR* Nonproliferative diabetic retinopathy, *PDR* Proliferative diabetic retinopathy

In the following the subfields, we observed some differences in CT between DME and non-DME patients within diabetes groups after age and sex adjustment in the SFCT (*P* = 0.019), N0.5 CT (*P* = 0.003), N1.5 CT (*P* < 0.001), central 1 mm CT (CCT) (*P* = 0.003), S3 CT (*P* = 0.012), I3 CT (*P* = 0.005), N3 CT (*P* < 0.001), T3 CT (P = 0.019), S6 CT (*P* = 0.004), and N6 CT (*p* = 0.001). CV(s) showed comparable results (*p* < 0.05). In all mentioned areas the CT(s) and CV(s) are less in the patients with DME. These amounts were far less than those in healthy control eyes (*p* < 0.05). Mean SFCT was 322.5 ± 62.2 μm in normal controls and 278.4 ± 65.6 μm in DME patients (*p* = 0.003). After age, sex, hypertension, hyperlipidemia, and FBS adjustment, considering collinearity, binary logistic regression analysis showed that DME was unrelated to the CT (in the model including all correlated factors in partial correlation analysis). However, hyperlipidemia was observed to be more significant (OR = 1.75; CI:0.77–3.98, *P* = 0.180) in the occurrence of DME in this model.

In the cases with diabetes, after adjusting for age and sex, the amount of FBS was correlated with N1.5 CT (*r* = − 0.205; *p* = 0.033), N3 CT (*r* = − 0.196; *p* = 0.042), S6 CT (*r* = − 0.284; *p* = 0.003), N6 CT (*r* = − 0.236; *p* = 0.014), I6 CT (*r* = − 0.236; *p* = 0.014), and T6 CT (*r* = − 0.190; *p* = 0.049). Similar concordant correlation was observed in the CV(s) as: N3 CV (*r* = − 0.1906; *p* = 0.048), S6 CV (*r* = − 0.269; *p* = 0.005), N6 CV (*r* = − 0.238; *p* = 0.013), I6 CV (*r* = − 0.215; *p* = 0.025), T6 CV (*r* = − 0.190; *p* = 0.048), total volume (*r* = − 0.238; *p* = 0.013). Multivariate linear regression analysis showed that FBS and DM duration had no association with the CT or CV.

### Correlation of FAZ with CT

FAZ at superficial (FAZs) and deep capillary plexus (FAZd) of macular area on enface images were automatically measured in each patient. The size of both FAZs and FAZd increased from the normal (0.28 ± 0.09 mm) to the NDR (0.33 ± 0.11 mm), NPDR (0.35 ± 0.18 mm) and then PDR (0.37 ± 0.10 mm) groups (*P* = 0.009). This increase was more prominent in the FAZd as normal (0.31 ± 0.08 mm) to the NDR (0.38 ± 0.18 mm), NPDR (0.50 ± 0.26 mm) and then PDR (0.52 ± 0.20 mm) groups (*P* < 0.001) (Table 5).

FAZs was correlated with hyperlipidemia (*r* = − 0.203; *p* = 035) and FAZd (*r* = 0.774; *p* < 0.001). None of the CT(s) and CV(s) associated with FAZ(s) after adjusting to the age and sex. In linear regression, after adjusting for age, sex, duration of DM, FBS, hypertension and hyperlipidemia, FAZs was significantly correlated with FAZd (beta = 0.532, *p* < 0.001). This means that in patients with diabetes, the FAZd will change 0.53 μm for each 1 μm of change in the FAZs.

FAZd was correlated with BCVA (*r* = − 0.351; *p* < 0.001), FAZs (*r* = 0.774; *p* < 0.001), CMT (*r* = 0.184; *p* = 0.057), S3 CT (*r* = − 0.208; *p* = 0.031), S3 CV (*r* = − 0.208; *p* = 0.031), S6 CT (*r* = − 0.29; *p* = 0.002), and S6 CV (*r* = − 0.249; *p* = 0.009). After multivariate linear regression analysis FAZd was associated with BCVA (B = -0.17, *p* = 0.006), FAZs (B = 0.768, *p* < 0.001), and CMT (B = 0.199, *p* = 0.001). No effect of CT on FAZ was shown in this study.

### Correlation of BCVA with CT

After controlling for age and sex, BCVA was correlated with FAZd (*r* = − 0.351; *p* < 0.001), CMT (*r* = − 0.266; *p* = 0.005), FT (*r* = − 0.273; *p* = 0.004), N0.5 CT (*r* = 0.233; *p* = 0.015), N1.5 CT (*r* = 0.271; *p* = 0.005), SFCT (*r* = 0.196; *p* = 0.042), S3 CT (*r* = 0.226; *p* = 0.019), N3 CT (*r* = 0.214; *p* = 0.026), I3 CT (*r* = 0.262; *p* = 0.006), T3 CT (*r* = 0.049; *p* = 0.190), S6 CT (*r* = 0.218; *p* = 0.024), N6 CT (*r* = 0.203; *p* = 0.035), and I6 CT (*r* = 0.2391; *p* = 0.013). Similar concordant correlation of BCVA was observed in the CV(s). In multivariate linear regression analysis only FAZd was linked to BCVA (B = − 0.255; *p* = 0.004), controlling for age, sex, length of DM, hyperlipidemia and hypertension. It means that for every micrometer increase in FAZd, BCVA (LogMAR) will decrease by 0.2. The CT was not correlated the BCVA in this research.

## Discussion

In the current study, we observed a declining order of CT from normal to NDR, then to NPDR. After that, CT increased in the PDR group, yet it never approached the normal level. We observed no significant differences in the CT, CMT and FT between normal and NDR groups. The CT and CV were not correlated with FBS, DM length, the occurrence of DME. BCVA and FAZ were not related to CT or CV.

According to the current study, it appears that the minimum CT in the patients with diabetes occurs in the NPDR. Thinner choroid may induce retinopathy on its own, or it may contribute to the advanced stages of retinopathy. CT studies in diabetes have resulted in divergent findings in the eyes with diabetic retinopathy [2, 10, 13–16].

Reduced oxygen supply of the outer layers of the retina and retinal pigment epithelium (RPE), may induce higher local VEGF concentration, resulting in changes in the diabetic choroid, that lead to DR. A continuous decrease in CT from the NDR to the NPDR phase in our study, supports past studies on the decrease in choroidal flow during DR. [10, 15] Laser Doppler flowmetry study found decreasing blood flow of the choroid in patients with type 2 diabetes before DR manifestation [1, 21]. ICG findings showed that vascular changes might affect the choroid in patients with NPDR [22]. EDI-OCT studies, including ours, showed that the CT was thinned in the NDR and mild, moderate, and severe NPDR stages [16, 17, 23, 24].

The mechanism of choroidal thinning in eyes with NDR and NPDR remains to be determined. Since choriocapillaris occupies a very limited portion of the whole choroid, attenuation of choriocapillaris alone cannot account for the change in CT observed in this study. However, this may be attributed to small and large vessel occlusion, resulting in hypoxia, atrophy, and remodeling, or it may be triggered by vascular autonomy disturbances in early DR.

Polymorphonuclear cells (PMNs) contribute a primary role in the pathogenesis of vaso-occlusive processes and endothelial cell injury in the diabetic choroid [4, 25, 26]. Increased Intercellular Adhesion Molecule 1 (ICAM-1) and P- selectin immunoreactivity in the diabetic choroidal vessels compared to subjects with no diabetes were already shown [6]. Recent study reported that capillary dropout is significantly increased in diabetic choroid compared with non-diabetic choroid using the alkaline phosphatase/nonspecific esterase (NSE/APase) choroidal preparations [27].

Choroidal infarction could explain the visual loss that occurs in patients with diabetes before retinopathy, because the choroidal vasculature is the source of nutrients and oxygen for photoreceptors and RPE [28]. Querques et al. [16] showed an overall thinning of the choroid in patients with diabetes. Xu et al. reported that the SFCT in patients with DM were slightly thicker, regardless of the presence or the stage of DR. However, of the 246 patients with diabetes mellitus in that population-based study, only 23 had DR [29].

After matching for axial length and age, Esmaeelpour et al. mapped CT in patients with both types of diabetes (I and II) and discovered that choroid was thinner in DM, regardless of disease stage, as compared to healthy controls [17, 23]. Vujosevic et al. investigated 102 patients with both forms of diabetes as well as 48 normal subjects and found that the mean SFCT progressively and significantly decreased with increasing stage of DR. There was no significant difference in SFCT between the controls and the eyes with NDR [24, 29].

We found that CT increased in the transition from NPDR to PDR, given the declining trend in the preceding stages. According to Kim et al., CT increased significantly as the disease progressed from NPDR to untreated PDR, which is consistent with our results [2].

Hypoxia could increase the expression of VEGF in RPE cells, pericytes, and microvascular endothelial cells, and could induce dysfunction of the blood-retinal barrier, which is supposed to be the basis of DR and DME in diabetes patients. In theory, choroidal thinning can account for increased susceptibility to retinal ischemia in DME patients due to insufficient choroidal blood flow [30].

In this study, DME occurrence was not affected from CT and CV. While a causality evaluation analysis is not currently scheduled, logistic regression revealed that hyperlipidemia has a greater relation with DME than CT decrease.

Nagaoka et al. demonstrated that NPDR patients with DME have lower choroidal flow using the laser Doppler velocity flowmetry technique than NPDR patients without DME [21]. On the other hand, some studies reported more pronounced CT in eyes exhibiting DME compared with eyes without DME, and no association between RT and CT was observed [2, 31]. The reported discrepancies may be attributed to various calculation techniques and the unadjusted analysis used in certain studies.

In agreement with other studies, DM duration had no significant correlations with CT(s) and CV(s) in our series [15, 22, 32]. According to our findings, FBS had no association with CT or CV. Jo and colleagues demonstrated that the choroid became significantly thicker 2 weeks after intensive diabetes control [33].

After controlling for conflicting variables, we observed no association between FAZ and CT. In Lee et al. study, no significant correlation between SFCT and FAZ size was found [34]. In this study, FAZs was more influenced by FAZd and vice versa. Interestingly, in diabetic patients, the FAZd will rise by 0.53 μm for every μm raise in FAZs.

The CT was not correlated with the BCVA in this study. After controlling for confounders, it is shown that for any micrometer increase in FAZd, BCVA (LogMAR) decreased by 0.2. Accordingly, Endo et al. reported no correlation between BCVA and CT in patients with DME having NPDR and PDR [35]. Eliwa et al. showed a significantly negative correlation between BCVA and central CT in the NPDR patients with DME group [13]. The discrepancies between the previous and current research may be attributed to the various levels of DR being studied as well as the previous studies’ uncontrolled design.

The strength of the study could be including three major groups of DR at the same time, recruiting naïve diabetic patient with more than 10 years duration of diabetes and adjusting for the common confounders in the analysis.

Our study had several limitations. The first is the relatively small number of the eyes in the individual subgroups. Furthermore, we measured the CT in both eyes of each participant, which is likely to influence the findings, even though the inter-eye similarity was tested before. Similarly, no correction for multiple comparisons and axial length is not provided.

Another problem is that manual segmentation of the chorioretinal and sclerochoroidal interfaces can result in errors. New framework for the analysis of 3D choroidal vessel networks has recently been presented, which enables highly repeatable vascular metrics to be used in future studies in DR. [36, 37] Though, manual annotation has been reported to have a segmentation accuracy of 96% [36]. In noisy choroidal images, selecting a suitable vessel segmentation scheme could be sometimes challenging.

The thickness of the choroid is thought to be a surrogate for choroidal blood flow, which accounts for 85% of all ocular blood flow, the impairment of which is thought to be associated with numerous ocular diseases such as AMD, diabetic retinopathy and central serous chorioretinopathy [38, 39].

Continuous thinning of the choroid during the course of diabetic retinopathy up to NPDR stage may herald progressive occlusive vasculopathy, which is interfered by a slight rise in CT in PDR phase that could be due to a rapid rise of VEGF. The lack of a relationship between CT and DME in this study, if replicated in future investigations, would be indicative of a lack of an association between choroid and retina in terms of VEGF production.

## Conclusions

Choroid was significantly thinner in DR patients compared to control participants. Choroidal thinning progresses in proportion to the severity of DR up to NPDR. The maximum decrease in CT occurred in the NPDR group in comparison with normal cases. CT and CV were not correlated with the occurrence of DME, FBS and DM duration. CT and CV had no association with BCVA and FAZ.

## Data Availability

**T**he datasets generated and/or analysed during the current study are not publicly available due to limitations of ethical approval involving the patient data and anonymity but are available from the corresponding author on reasonable request.
